# Control of the meiotic cell division program in plants

**DOI:** 10.1007/s00497-013-0223-x

**Published:** 2013-07-14

**Authors:** Erik Wijnker, Arp Schnittger

**Affiliations:** 1 Department of Molecular Mechanisms of Phenotypic Plasticity, Institut de Biologie Moléculaire des Plantes du Centre National de la Recherche Scientifique, Université de Strasbourg, 67084 Strasbourg, France; 2 Trinationales Institut für Pflanzenforschung, Institut de Biologie Moléculaire des Plantes du Centre National de la Recherche Scientifique, Université de Strasbourg, 67084 Strasbourg, France

**Keywords:** Arabidopsis, Meiosis, Cell cycle, Cyclin-dependent kinase, Cyclin, Recombination

## Abstract

While the question of why organisms reproduce sexually is still a matter of controversy, it is clear that the foundation of sexual reproduction is the formation of gametes with half the genomic DNA content of a somatic cell. This reduction in genomic content is accomplished through meiosis that, in contrast to mitosis, comprises two subsequent chromosome segregation steps without an intervening S phase. In addition, meiosis generates new allele combinations through the compilation of new sets of homologous chromosomes and the reciprocal exchange of chromatid segments between homologues. Progression through meiosis relies on many of the same, or at least homologous, cell cycle regulators that act in mitosis, e.g., cyclin-dependent kinases and the anaphase-promoting complex/cyclosome. However, these mitotic control factors are often differentially regulated in meiosis. In addition, several meiosis-specific cell cycle genes have been identified. We here review the increasing knowledge on meiotic cell cycle control in plants. Interestingly, plants appear to have relaxed cell cycle checkpoints in meiosis in comparison with animals and yeast and many cell cycle mutants are viable. This makes plants powerful models to study meiotic progression and allows unique modifications to their meiotic program to develop new plant-breeding strategies.

## Breaking the rules of mitosis

**Fig. 1:**
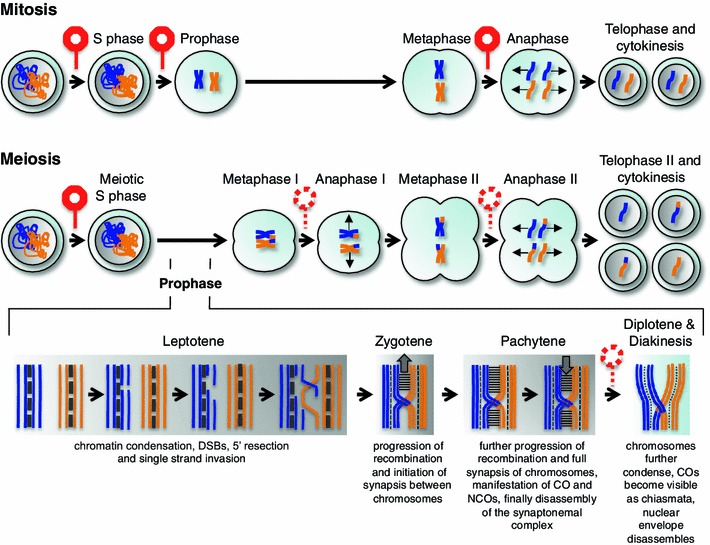
Overview of a mitotic and meiotic division. *Top panel* major transitions in the mitotic cell cycle. Only one pair of homologous chromosomes is shown in *orange* and *blue*, with each *line* representing one chromatid. Chromatids duplicate during S phase, condense at prophase and segregate at anaphase followed by decondensation. Note the absence of the nuclear envelope during mitosis. The *middle panel* concurrent meiotic stages, with the first meiotic division added onto the mitotic program. Note that meiosis I is unique in segregating homologous chromosomes instead of chromatids. The segregation of sister chromatids at anaphase II resembles a mitotic division. The *lower panel* highlights different stages of the meiotic prophase; the events at the recombination sites are largely simplified, for a more detailed description see other reviews on this topic (Edlinger and Schlogelhofer 2011; Osman et al. 2011). Please note that the leptotene stage shows the highest level of magnification, zygotene/pachytene is intermediate and diplotene/diakinesis shows the lowest magnification. Single *blue* and *orange lines* in this panel indicate single DNA strands, and two adjacent *lines* represent one chromatid. Double-strand breaks (DSBs) in leptotene comprise the first steps of homologous recombination. Three mitotic checkpoints are highlighted with *red signs*. Meiosis in plants presumably shares one checkpoint at the beginning of meiotic S phase with the one found in animals and yeast (*in red*), whereas other meiotic checkpoints known from animals and yeast appear to be not present or function in a relaxed manner in plants (signs in *red dashed lines*)

**Fig. 2:**
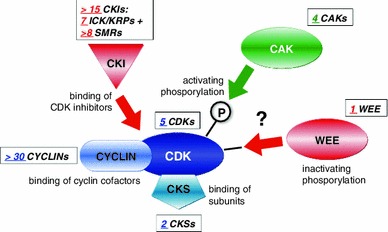
Overview over the core cell cycle machinery in Arabidopsis. Progression through mitosis and meiosis is promoted by the activity of CDKs who require for full activity the binding of cyclin partners. These heterodimers can be regulated at multiple levels, e.g., binding of other subunits, CDK inhibitors and activating phosphorylation. The role of the inhibitory phosphorylation that is mediated by Wee1-type kinases in yeast and animals is not very well understood in plants and appears to be used in a different context than in other species. Analysis of cell cycle regulators is challenging in plants through the relatively high number of family members that often act at least partially redundantly. Here, the family sizes of the core cell cycle machinery components are given for Arabidopsis

While the reasons why meiosis and sex have evolved are under debate (see review and hypothesis by Hoerandl and Hadacek in this issue of Plant Reproduction), it is widely accepted that meiosis was derived from mitosis (Wilkins and Holliday 2009; Cavalier-Smith 2010). Progression through mitosis, which typically generates two daughter cells that are genetically identical to the mother cell, is controlled by the activity of cyclin-dependent kinases (CDKs) (Morgan 1997) (Figs. 1, 2). Although the control machinery displays many species-specific variations and is subject to adaptation, the general principle of CDK-driven progression through mitosis appears to be conserved from humans to plants (Harashima et al. 2013).

In contrast to mitosis, meiosis does not generate genetic copies of the mother cell; instead, the nuclear DNA content is halved when two subsequent chromosome segregation events immediately follow one another without an intermittent S phase (Fig. 1). The first meiotic division is crucially different from a mitotic division since homologous chromosomes instead of sister chromatids segregate to opposite poles. Importantly, the first meiotic division generates new allele combinations. These result both from meiotic crossovers, i.e., the reciprocal exchange of DNA segments between homologues after repair of deliberately induced double-strand DNA breaks at prophase I, and from random homologue segregation at anaphase I (Fig. 1). During the second meiotic division, sister chromatids segregate, similar to a mitotic division (Fig. 1) (Brar and Amon 2008). To allow for the unique segregation of homologues during meiosis I, a special segregation machinery is present. This ensures that centromeric cohesion between the two sister chromatids of a chromosome is maintained during anaphase I and that their kinetochores are mono-oriented toward the same cell pole. Sister chromatid cohesion is then only completely lost in anaphase II when the kinetochores of both sister chromatids are attached to opposite spindles.

In spite of these differences, entry and progression through meiosis are controlled by many of the same regulators as in mitosis, i.e., CDK-cyclin complexes and the anaphase-promoting complex/cyclosome (APC/C) (Pesin and Orr-Weaver 2008; Cooper and Strich 2011). Thus, a key question is how these complexes and activities are reprogrammed and adapted in meiosis to promote events that are strictly prohibited in a mitotic cycle and how advancement in meiosis is coordinated with recombination and chromosome distribution.

The focus of this review is on the cell cycle control aspect of meiosis in flowering plants. Excellent reviews on other aspects of plant meiosis, especially recombination, have been published elsewhere (Edlinger and Schlogelhofer 2011; Ma 2006; Osman et al. 2011; Mercier and Grelon 2008). After an introduction on the initiation and exit of the meiotic program, we summarize recent attempts to complete the parts list of meiotic cell cycle control machinery in plants. Focus will be on the function and regulation of CDK-cyclin complexes and the APC/C as major driving forces of meiosis. Finally, we summarize how the specialties of the plant meiotic program can be exploited in plant breeding.

## Starting the meiotic program

Most plants, like the majority of all eukaryotes, reproduce sexually. During fertilization, the gametes from each parent fuse to give rise to a zygote from which the new organism develops. To prevent genome doubling in every new generation, the DNA content of the gametes has to be reduced through meiosis. Conversely, halving of chromosome number is strictly limited to occur only during gamete formation. Thus, special programs must exist to specify meiotic cells and to precisely control the entry as well as exit from the meiotic division program.

In animals, meiosis is executed by germ line cells that are separated from somatic cells during early embryogenesis (Wylie 1999). In flowering plants, however, meiotic cells, i.e., megaspore mother cells (by definition the female side) and microspore mother cells (male), are formed late during development, i.e., a subepidermal layer of archesporial cells differentiates into microspore mother cells in anther primordia, while megaspore mother cells differentiate from a single subepidermal cell in the tips of ovule primordia (Grossniklaus and Schneitz 1998; Goldberg et al. 1993). Substantial work over the last years has led to the identification of genetic pathways that are required for a meiotic cell fate. In case of the megaspore mother cell, this pathway includes the nuclear-localized protein SPOROCYTELESS/NOZZLE (SPL/NZZ) (Yang et al. 1999; Balasubramanian and Schneitz 2000) and the further downstream-acting homeobox transcription factor WUSCHEL (WUS) (Lieber et al. 2011; Groß-Hardt et al. 2002). WUS is required for the expression of two redundantly acting genes *WINDHOSE 1* (*WIH1*) und *WIH2* that encode novel peptides whose absence leads to the loss of a morphological distinguishable functional megaspore mother cell (Lieber et al. 2011). Possible receptors for the WIH peptides are the tetraspanin-type transmembrane protein TORNADO 2 (TRN2) and the leucine-rich repeat protein TRN1 (Lieber et al. 2011). However, WIH-TRN interactions still need to be confirmed, and it is also up to now not clear what the downstream targets of this putative signaling cascade are.

It has been found that in particular posttranscriptional regulation, i.e., at the RNA level, is important for germ cell specification. The identification of several mutants in the *ARGONAUTE* (*AGO*) family in Arabidopsis, maize and rice implicated small RNAs in regulating meiotic progression, the repression of germ cell fate in somatic tissues, or, as was shown in rice, by repressing a somatic fate in germ cells (Nonomura et al. 2007; Olmedo-Monfil et al. 2010; Singh et al. 2011). The importance of posttranscriptional regulation for megaspore mother cell fate specification has been further underlined by the identification of the *SUPPRESSOR OF GENE SILENCING 3* (*SGS3*) and of *RNA*-*DEPENDENT RNA POLYMERASE 6* (*RDR6*) (Olmedo-Monfil et al. 2010) whose mutations, like *ago9* in Arabidopsis, lead to multiple megaspore mother cells and function in the biogenesis of double-stranded RNA. Another possible link to posttranscriptional control of meiocyte fate comes from the analysis of *MEIOSIS ARRESTED AT LEPTOTENE 2* (*MEL2*) in rice that encodes for protein with a RNA recognition motif. Loss of MEL2 function results in a failure of most meiocytes to enter meiotic S phase. The few cells that proceed to prophase arrest at early stages and show perturbed meiotic characteristics (Nonomura et al. 2011).

Other genes that affect meiocyte specification are not yet functionally understood but are possibly linked to transcriptional and/or posttranscriptional control. For instance, the mutation of two DNA methyltransferases in maize leads to unreduced gametes and multiple embryo sacs, exemplifying control at the chromatin level of both female and male meiosis (Garcia-Aguilar et al. 2010). *AMEIOTIC 1* (*AM1*) encodes an unknown protein from maize, and most *am1* alleles lack all indications of meiotic prophase, suggesting a key role in establishing a meiotic program (Pawlowski et al. 2009). In one *am1* allele, however, prophase is apparently initiated but cells arrest in leptotene/zygotene stage (Pawlowski et al. 2009; Golubovskaya et al. 1993). The phenotype of mutants in a rice *AM1* homologue resembles the one seen in this later allele from maize, also resulting in meiotic arrest during early prophase (Che et al. 2011). In contrast to maize and rice, mutants in the closest homologue of *AM1* in Arabidopsis-designated *DYAD*/*SWITCH 1* (*SWI1*) complete a meiotic program and give rise to viable gametes (Mercier et al. 2001; Agashe et al. 2002; Siddiqi et al. 2000; Motamayor et al. 2000). Nevertheless, *dyad*/*swi1* show some similarities with *am1* since alleles were described in which female, but remarkably not male meiosis shows mitotic-like divisions resulting from a failure to undergo synapsis followed by an equational division in which sister chromatids segregate. Male meiosis is either unaffected, or shows the complete loss of sister chromatid cohesion during meiotic prophase (Motamayor et al. 2000; Agashe et al. 2002; Mercier et al. 2001). Thus, it is likely that AM1-type proteins have undergone species-specific diversification and/or are involved in many different processes including the entry into meiosis.

Currently, it is not clear how these developmental regulators of meiotic cell fate initiate the meiotic cell division program. Programming of meiosis already starts before or during the meiotic S phase. An indication for this is the observation that in most if not all organisms meiotic S phase is much longer than an S phase preceding mitosis (Bennett and Smith 1972; Holm 1977). Specialties of meiotic S phase include the loading of a meiosis-specific cohesion complex, including the RADIATION SENSITIVE 21 (RAD21)-family protein RECOMBINATION DEFICIENT 8 (REC8), onto chromatin that ensures sister chromatid cohesion throughout meiosis I.

Similar to the entry, also the exit from a meiotic program needs to be strictly controlled to maintain genome stability and gene dosage. This is especially important in plants since the spores generated after meiosis will undergo a few (in flowering plants) up to many mitotic divisions (in moss) during the gametophytic life phase. Remarkably, mutants for the Arabidopsis gene *THREE DIVISION MUTANT 1* (*TDM1*)/*MALE STERILE 5* (*MS5*)/*POLLENLESS 3* that encodes a protein with a yet unknown function undergo a third meiotic division without intervening S phase, indicating a failure in shutting down the meiotic program (Ross et al. 1997; Sanders et al. 1999; Glover et al. 1998). Similar problems in meiotic exit were described for mutants in the cyclin-dependent kinase inhibitor *Roughex* in Drosophila (Gönczy et al. 1994; Foley and Sprenger 2001). The observation that a *tdm1* phenotype can be phenocopied by expressing a non-degradable version of the meiotic A-type cyclin TARDY ASYNCHRONOUS MEIOSIS (TAM, see below) argues that TDM1 may also act in some way to restrict meiotic CDK activity (Cromer et al. 2012). However, while accumulating evidence also from plants indicates that meiotic exit is coupled to reduced CDK activity (see below), it is not clear yet how high CDK activity could possibly induce a third meiotic division.

## Progression through meiosis: the role of CDK-cyclin complexes

**Fig. 3:**
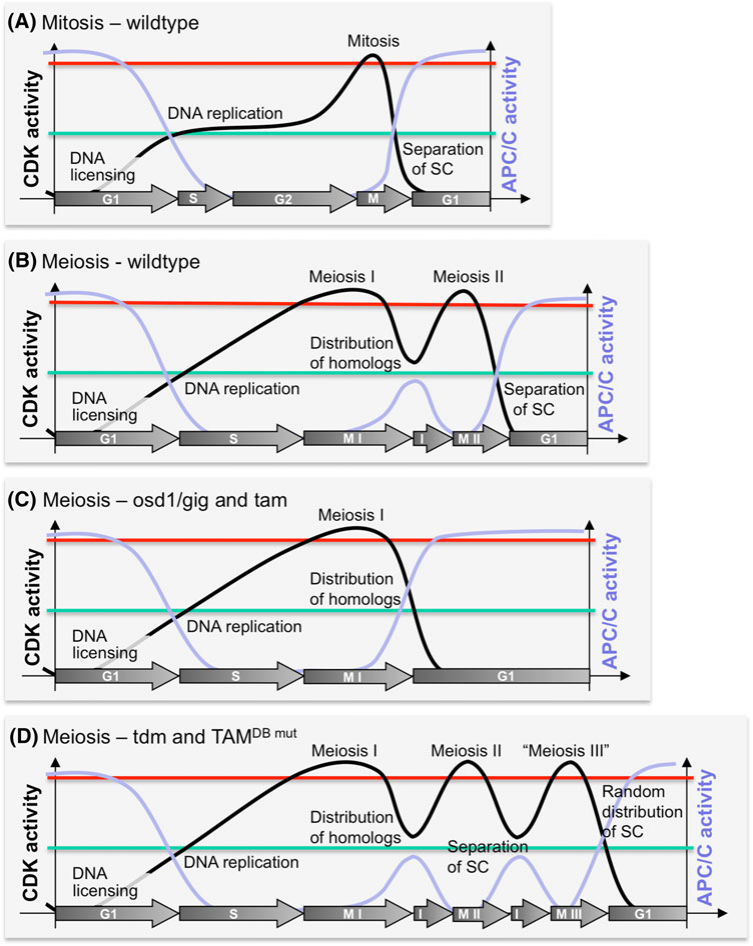
Hypothetical activity levels of CDK and APC/C complexes during mitosis and meiosis. **a** Progression through mitosis is thought to rely on increasing levels of CDK activity (*black line*). Medium levels of CDK activity are required for the induction of S phase, and high levels are necessary to promote M phase. Putative threshold levels for S phase are indicated by a *horizontal green line*, threshold concentrations for M phase by a *red line*. Please note that most likely CDK activity in plants is separated into S phase CDK-cyclin levels and M phase CDK-cyclin levels that are for simplicity reasons not separately shown here. In order to license the origins of replication for S phase, CDK activity as to be low. This is largely accomplished by the activity of the APC/C (*indigo line*) that mediates the degradation of cyclins at the end of mitosis and thus sets back CDK activity. APC/C^CDC20^ requires phosphorylation by CDK-cyclin complexes for activity but is kept largely inactive until anaphase. This inhibition will only be released if all chromosomes are attached to the mitotic spindle. The APC/C mediates then the degradation of securin which liberates separase that in turn cleaves the centromeric cohesions between sister chromatids (SC) to allow their subsequent segregation. After degradation of cyclins and drop of CDK activity, the APC/C is kept active by the Cdh1/Fzr/CCS52 adaptor protein. **b** During the meiotic S phase that typically takes much longer than a mitotic S phase, chromosomes are prepared for meiosis, for instance by the incorporation of the meiosis-specific cohesion *REC8*. Prophase I immediately starts after S phase (see also Fig. 1) that again typically takes much longer than the mitotic prophase. Dampening of APC/C activity and/or maintenance of CDK activity after anaphase I is crucial to prevent exit from meiosis and to establish interkinesis (the short phase between meiosis I and II) before meiosis II. To what level CDK and APC/C activities are changed is purely speculative in the graph. **c** The second meiotic division is skipped in mutants like osd1/gig and tam. Presumably, loss of TAM directly reduces CDK activity levels, while loss of OSD1 leads to full activation of the APC/C and hence a drop in CDK activity via degradation of meiotic cyclins. **d** Mutants in TDM and plants expressing a TAM mutant version in which the recognition sequence for the APC/C (*destruction box*) is mutated enter a third meiotic division in which then the sister chromatids are randomly distributed. It is plausible that such a third division, similar to the first and second division, is guided by raising and falling levels of CDK and APC/C activities. Mutants in TAM also slow down the progression of meiotic Prophase I, a feature that is not covered here

Progression through the mitotic cell cycle has been found to rely on quantitative and qualitative aspects of CDK-cyclin complexes (Fig. 3a). On the one hand, it has been found that oscillating levels of kinase activity drive the advancement in the cell cycle—a major regulator of this oscillation is the APC/C (see next chapter). For a cell to enter the DNA replication phase (S phase) from a preceding gap phase 1 (G1), the CDK-cyclin activity has to reach a certain threshold level. Then, a higher level of kinase activity is required for a cell to move from gap phase 2 (G2) that follows S phase into mitosis (M phase). After mitosis, CDK activity drops which is required for new licensing of replication origins as a prerequisite for another S phase (Nasmyth 1996; Stern and Nurse 1996). These oscillations are thought to coordinate the different cell cycle events and promote a unidirectional progression in the cell cycle, e.g., by preventing untimely re-replication of the nuclear DNA before mitosis. Experimental evidence for this hypothesis has recently been provided by fission yeast cells that were engineered to have a chemically tunable CDK-cyclin complex (Coudreuse and Nurse 2010).

Cyclin-dependent kinases are regulated at multiple levels, and a key determinant of CDK activity is the amount and type of cyclin partners that are available (Pines 1995) (Fig. 2). There are typically S- and M-phase CDKs and cyclins. The general picture in animals is that D-type and E-type cyclins promote entry into S phase, while cyclin A controls S phase as well as early mitotic events, and B-type cyclins control mitosis (Pines 1995). Importantly, different CDK-cyclin complexes were found to have different, although sometimes partially overlapping, substrate specificities (Pagliuca et al. 2011). It is tempting to speculate that these specificities also contribute to the orchestration of the cell cycle.

In plants, clear homologues for A- and B-type cyclins have been found next to a cyclin class that has been named D-type cyclins but is equidistant to animal D- and E-type cyclins (Wang et al. 2004a). Similar to the situation in animals, different CDK-cyclin complexes appear to have distinct activity levels against different substrates (Harashima and Schnittger 2012; Nowack et al. 2012). Notably, many of the key components involved in recombination both in animals and plants harbor consensus CDK phosphorylation sites and/or cyclin-binding signatures, e.g., DISRUPTED MEIOTIC cDNA 1 (DMC1), REC8 and SPORULATION-DEFICIENT 11 (SPO11) (Esposito and Esposito 1969; Bishop et al. 1992; Ponticelli and Smith 1989), suggesting that not only the general progression through meiosis but also the meiosis-specific recombination events are orchestrated by CDK-cyclin complexes.

One complication to study cell cycle control in plants is the large number of some of the cell cycle regulators present in the genome. For instance, next to at least five central cell cycle CDKs (CDKA;1, CDKB1;1, CDKB1;2, CDKB2;1 and CDKB2;2), there are more than 30 cyclins in Arabidopsis (Fig. 2) (Wang et al. 2004a; Vandepoele et al. 2002). Thus, an obvious first question is which cell cycle regulators are involved to drive plant meiosis.

Arabidopsis CDKA;1, which shows the highest level of similarity with Cdk1 and Cdk2 among the animal CDKs, appears to be predominantly involved in controlling S phase entry next to its role in mitosis (Nowack et al. 2012). Homozygous *cdka;1* mutants are viable but severely compromised, precluding clear developmental analyses (Nowack et al. 2012). Weak loss-of-function alleles of *CDKA;1* were found to be completely sterile, and morphological analysis of male meiosis and gametogenesis indicated a central role in meiosis (Dissmeyer et al. 2007, 2009). A key role of CDKA;1 in meiosis is further supported by the immunolocalization of CDKA;1 during meiosis as well as the detection of functional CDKA;1-YFP fusion proteins in meiocytes (Bulankova et al. 2010; Zhao et al. 2012).

Interestingly, an important chromosome pairing regulator of wheat, *Pairing homeologous 1* (*Ph1*), was proposed to be an epiallele of a wheat *CDKA;1* homolog. The presence of *Ph1* prevents the recombination between the three homoeologous genomes (A, B and D) that are present in bred wheat and thus is a crucial determinant of wheat fertility (Riley and Chapman 1958). The *Ph1* locus was mapped to a region of approximately 2 Mb on chromosome 5B (Al-Kaff et al. 2008; Griffiths et al. 2006). In the center of this region lies a heterochromatic region, apparently translocated from chromosome 3A. Flanking this heterochromatic region resides a cluster of pseudo genes that show similarities to *CDKA;1*. It has been proposed that the heterochromatic region stimulates the production of small RNA specimens from the pseudo *CDK* genes leading in turn to a down-regulation of the expression of endogenous *CDKA*-like genes (Griffiths et al. 2006). Further support for this hypothesis comes from the observation that removal of *Ph1* leads to a transcriptional up-regulation of *CDKA*-like genes on other chromosomes (Al-Kaff et al. 2008). CDK action is counter balanced by phosphatases (Fisher et al. 2012), and interestingly, treatment of wheat plants with okadaic acid, a phosphatase inhibitor, led to pairing of homeologous chromosomes resembling the loss of *Ph1* (Knight et al. 2010; Greer et al. 2012). However, the mapping interval of *Ph1* is still large, and in the absence of direct functional evidence, it is currently not unambiguously clear whether *Ph1* encodes an epiallele of the wheat *CDKA* kinase(s). Even if so, it is not at all understood how down-regulation of CDK activity could accomplish the complex phenotypes seen in *Ph1*.

B1-type CDKs appear to function mostly in mitotic entry but have also some function in S phase (Nowack et al. 2012; Vanneste et al. 2011; Xie et al. 2010). The full fertility of the double mutant *cdkb1;1 and cdkb1;2* indicates only a minor or redundant role of B1-type CDKs in meiosis. B2-type CDKs were found to accumulate specifically in M phase (Menges et al. 2005). Both, the down-regulation by an RNAi approach as well as their overexpression resulted in severely compromised plants with malfunctioning shoot apical meristem precluding a judgment of a role in meiosis (Andersen et al. 2008). Given the similarities of the second meiotic division with mitosis (Fig. 1), it is at least tempting to speculate that CDKB2s have a role in meiosis II.

The first two cyclins in Arabidopsis that were shown to have a meiotic function are the A-type cyclin TAM, also called CYCA1;2, and SOLO DANCERS (SDS), an atypical cyclin that shows similarities with A- and B-type cyclins (Table 1). In weak *tam* mutants, progression through meiosis I and II is slowed down, hence the name. Null mutants in *TAM* exit the meiotic program after the first division and produce diploid gametes (Fig. 3c) (Magnard et al. 2001; Wang et al. 2004b; d’Erfurth et al. 2010; Bulankova et al. 2010). Although TAM is not only expressed in meiosis, its meiotic function appears to be specific among the A1-type cyclins since it was found to be the only A1-type cyclin expressed in meiosis (Bulankova et al. 2013). Consistently, mutants in the closely related *CYCA1;1* did not show any meiotic aberrations and even double mutants between *cyca1;1* and *tam* did not enhance the *tam* mutant phenotype (Cromer et al. 2012). TAM acts in a not yet fully understood genetic network with two other meiotic genes: *SUPPRESSOR WITH MORPHOGENETIC EFFECTS ON GENITALIA 7* (*SMG7)* and *TDM1* (see sections on meiotic entry and meiotic checkpoints). While loss of SMG7 results in arrest at anaphase II, the double mutant *tam smg7* also progresses till this arrest point, i.e., the effect of *tam* can be suppressed by mutations in *SMG7* (Bulankova et al. 2010). Similarly, unexpected was the finding that the *tdm1* mutant phenotype, resulting in a third meiotic division, is epistatic to the arrest after the first meiotic division seen in *tam* (Bulankova et al. 2010). Finally, meiosis in a *smg7 tdm1* double mutant was found to overcome the *smg7* arrest point and normally progress to telophase II where after it even entered a third meiotic division as seen in *tdm1* mutants. These genetic analyses link TDM1 and SMG7 to the regulation of CDK activity.

**Table 1:** Synopsis of meiotic cell division regulators in Arabidopsis

Protein class	Name	Function	References
CDK	CDKA;1	Homologue of yeast Cdc2/Cdc28 combining functional elements of human Cdk1 and Cdk2, characterized by PSTAIRE signature in the cyclin- binding domain; present throughout meiosis, localizes in particular to the organellar band that separates the two cell poles after meiosis I; essential for meiosis, in particular high kinase activity appears to be important to prevent premature exit from meiosis I; builds active complex with SDS and TAM; also expressed in somatic cells	(Dissmeyer et al. 2007, 2009; Cromer et al. 2012; Bulankova et al. 2010; Harashima and Schnittger 2012; Nowack et al. 2012; Zhao et al. 2012)
CYCLIN	CYCA2;1	Like TAM expressed from leptotene to pachytene; also expressed in somatic cells	(Bulankova et al. 2013)
	CYCA2;2	Present in leptotene, localized to nuclei; also expressed in somatic cells; mutants do not show meiotic defects; however, the triple mutant with *cyca2;3* and *cyca2;4* has defects in chromosome condensation and segregation	(Bulankova et al. 2013; Vanneste et al. 2011)
	CYCA2;3	Promoter reporter lines suggest no expression during meiosis; however, the triple mutant with *cyca2;2* and *cyca2;4* has defects in chromosome condensation and segregation	(Bulankova et al. 2013; Vanneste et al. 2011)
	CYCA2;4	Promoter reporter lines suggest no expression during meiosis; however, the triple mutant with *cyca2;2* and *cyca2;3* has defects in chromosome condensation and segregation	(Bulankova et al. 2013; Vanneste et al. 2011)
	CYCA3;2	Present in leptotene, localized in nuclei, also expressed in somatic cells; mutants do not show meiotic defects	(Bulankova et al. 2013)
	CYCA3;3	Specifically expressed in meiosis and present throughout meiosis I and II; no obvious destruction box; mutants do not show meiotic defects	(Bulankova et al. 2013)
	CYCA3;4	Present in leptotene, localized in nuclei, also expressed in somatic cells; mutants do not show meiotic defects	(Bulankova et al. 2013)
	CYCB3;1	The only B-type cyclin detected in meiosis based on promoter reporter lines, present from zygotene to metaphase I, where it localizes to the spindle, reappears in metaphase II where it again localizes to the spindle; also expressed in somatic cells, CYCB3;1 inhibits precocious cell wall formation in meiosis redundantly with SDS	(Bulankova et al. 2013)
	SDS	Atypical meiosis-specific cyclin that displays similarities with A- and B-type cyclin; expressed throughout meiosis and no obvious destruction box found; *sds* mutants display defects in homologue pairing and crossover formation during prophase I, leading to greatly reduced levels of meiotic recombination; SDS protein interacts with both CDKA;1 and CDKB1;1 in yeast two-hybrid interaction assays but has only high kinase activity with CDKA;1 in vitro; ectopically positioned cell walls in low percentage of *sds* mutants that are strongly enhanced in double mutants with *cycb3;1*	(Azumi et al. 2002; Chang et al. 2009; De Muyt et al. 2009; Bulankova et al. 2013; Harashima and Schnittger 2012)
	TAM (CYCA1;2)	Expressed in meiotic prophase from leptotene to pachytene, similar to *CYCA2;1*, required for fast progression through male meiosis I and II; mutant phenotype can be suppressed by mutations in SMG7 or TDM1; no meiotic function has been found for the closely related CYCA1;1, and double mutants between *cyca1;1* and *tam* do not show an enhanced tam mutant phenotype; also expressed in somatic cells	(Magnard et al. 2001; Wang et al. 2004b; d’Erfurth et al. 2010; Bulankova et al. 2010; Bulankova et al. 2013)
APC/C inhibitor	OSD1 (GIG)	Expression not determined; mutants exit the meiotic program after meiosis I; mutants develop cells with increased DNA content through endomitosis in vegetative tissues; interacts with the APC/C coactivators CDC20 and CCS52A1; genetic evidence from vegetative cells indicates an inhibitory function in particular for CDC20; can be phosphorylated by CDKA;1-TAM complexes in vitro	(d’Erfurth et al. 2009; Cromer et al. 2012; Iwata et al. 2011)
TPR-repeat domain protein with a 14-3-3 domain	SMG7	Expression in meiosis not clear; SMG7 is involved in Nonsense-Mediated RNA decay (NMD); mutants have pleiotropic phenotypes caused by an autoimmune-like response; in meiosis, loss of SMG7 results in an arrest after anaphase II; appears to act in the same genetic pathway as TDM1	(Bulankova et al. 2010; Riehs-Kearnan et al. 2012; Riehs et al. 2008)
Plant-specific protein with unknown function	TDM 1 (MS5, POLLEN-LESS3)	Expression in meiosis not clear; represses a third meiotic division through an unknown mechanism; is epistatic to mutants in TAM and SMG7	(Sanders et al. 1999; Bulankova et al. 2010; Ross et al. 1997; Glover et al. 1998)

The atypical cyclin SDS is specifically expressed in meiosis, and *sds* mutants display defects in homologue pairing and formation of crossovers during prophase I, leading to greatly reduced levels of meiotic recombination (Azumi et al. 2002; Chang et al. 2009; De Muyt et al. 2009; Bulankova et al. 2013). SDS activity is required for the recruitment of the recombinase DMC1 to chromosomes (De Muyt et al. 2009). SDS has been found to interact with both CDKA;1 and CDKB1;1 in yeast two-hybrid interaction assays (Azumi et al. 2002), but in in vitro kinase assays, SDS showed high activity only in conjunction with CDKA;1 (Harashima and Schnittger 2012).

Recently, all A- and B-type cyclins of Arabidopsis were assessed for expression during meiosis, and distinct meiotic accumulation patterns were found for eight of them providing a framework for further studies (Table 1) (Bulankova et al. 2013). CYCA2;2, CYCA3;2; CYCA3;3, CYC3;4 and SDS were detected already early in Prophase. In mid Prophase, besides TAM also CYCA2;1 appeared while CYCA2;2, CYCA3;2 and CYCA3;4 disappeared. CYCB3;1 specifically accumulated in Metaphase I and Metaphase II and localized to the spindle. Next to SDS, only CYCA3;3 was found to be present throughout meiosis. However, no mutant phenotype was found in *cyca3;3* mutants and similarly no perturbation of the meiotic program was found in *cyca3;2* or *cyca3;4* mutants. However, a redundant function of the three meiotically expressed A3-type cyclins cannot be ruled out at the moment (Bulankova et al. 2013).

Unexpectedly, given its localization to the spindle in late meiosis I and II, mutants in *CYCB3;1*, the only B-type cyclin that was found to be expressed in meiosis, showed the formation of cell wall-like structures from prophase I through entire meiosis (Bulankova et al. 2013). A closer examination revealed that ectopically positioned cell walls are also formed in low percentage of *sds* mutants and indeed, introgression of *sds* into the *cycb3;1* mutant background strongly enhanced this phenotype. Thus, SDS and CDKB3;1 appear to have multiple and likely not connected roles during meiosis; first in orchestrating recombination (SDS) and presumably spindle formation (CDKB3;1) and second in contributing to general kinase levels preventing the formation of premature cytokinesis.

The question as to which cyclins are involved in meiosis is likely more complex than suggested by the above-mentioned expression patterns. For instance, both *CYCA2;3* and *CYCA2;4* were not found to be present in meiosis, and mutants in *CYCA2;1* and *CYCA2;2* were fully fertile. However, the triple mutant *cyca2;2 cyca2;3 cyca2;4* shows defects in chromosome condensation and segregation, arguing either for low expression/masked detection of CYCA2;3 and/or CYCA2;4 or for a complex compensatory interaction in which normally not expressed A2-type cyclins become up-regulated in a single-mutant background (Bulankova et al. 2013). Nonetheless, the very defined expression and localization patterns of the different cyclins argues that, instead of one single central regulator, many distinct CDK-cyclin dimers with partially overlapping functions drive progression through plant meiosis.

## Progression through meiosis: APC/C control

The APC/C, whose activity is coupled to CDK action, is one of the most important regulators of the mitotic cell cycle. It is a large 1.5 MDa multi-subunit E3 ubiquitin ligase that marks target proteins for degradation by the proteasome. The two major functions of the APC/C are the mediation of the turnover of cyclins and securin, an inhibitor of separase that cleaves the centromeric cohesion of sister chromatids and by that promotes the progression of Anaphase. These functions appear to be conserved in all eukaryotes although the APC/C might have been additionally recruited for developmental roles in a species-specific manner (Marrocco et al. 2010; Peters 2006; Heyman and De Veylder 2012).

In a simplified view, high CDK levels promote APC/C activity by phosphorylating several components, among them CDC20, a WD40 repeat-containing coactivator, that is important for the recognition of substrates such as B-type cyclins (Pesin and Orr-Weaver 2008). Through the action of the spindle checkpoint (see also next section), the APC/C largely remains inactive until all sister chromatids are attached to the mitotic spindle and under tension indicating an equal alignment of the chromosomes in the metaphase plate and allowing subsequently their equal distribution (Jia et al. 2013; Lara-Gonzalez et al. 2012). The activated APC/C eliminates kinase activity through degradation of cyclins (Fig. 3). This, in turn, also shuts down APC/C^CDC20^ activity itself. However, there is a related coactivator protein for the APC/C, Cdh1/Fzr, known as CELL CYCLE SWITCH PROTEIN 52 (CCS52) in plants (Cebolla et al. 1999), that functions at low CDK levels and maintains APC/C activity during the exit from mitosis and in G1 (Fig. 3) (Peters 2006).

In animals and yeast, the APC/C was found to be required for both meiosis I and meiosis II (Cooper and Strich 2011). For the progression from metaphase I to anaphase I, the APC/C needs to degrade the separase inhibitor securin, like it does in mitosis. Separase then cleaves the meiotic cohesin REC8 along the chromosome arms, allowing the resolution of chiasmata between homologous chromosomes and their segregation to opposite poles. However, REC8, protected by Shugoshin (Kitajima et al. 2004), is not destroyed in the centromeric regions because of which the two sister chromatids of each homologous chromosome remain connected. Through different mechanisms, the kinetochores of both sister chromatids are oriented in the same direction or reduced to only one functional kinetochore. The centromeric REC8 is finally cleaved in meiosis II, allowing the separation of sister chromatids, resembling the situation in mitosis (Brar and Amon 2008).

As outlined above, the activation of the APC/C in mitosis results in a drop of CDK activity and initiates a mitotic exit program. Studies in animals and yeast have shown that CDK activity is kept high after meiosis I by dampening APC/C-mediated proteolysis and by increased synthesis of meiotic cyclins (Hochegger et al. 2001; Izawa et al. 2005; Borgne et al. 2002; Gross et al. 2000). The requirement for high CDK activity to prevent cytokinesis after Arabidopsis meiosis I is highlighted by the phenotypes of *tam* null mutants that terminate meiosis after the first division and hypomorphic *cdka;1* mutants that also appeared to make a cell wall after only one division (Dissmeyer et al. 2007; Bulankova et al. 2010; d’Erfurth et al. 2010). Consistently, active CDKA;1 complexes were detected at the organellar bands that separate the two cell poles after meiosis I where they may prevent cytokinesis (Bulankova et al. 2010).

Furthermore, there is evidence that the maintenance of elevated CDK activity after meiosis I is linked to the regulation of APC/C in Arabidopsis similar to the situation in animals; since, mutants in an APC/C inhibitor protein called OMISSION OF SECOND DIVISION 1 (OSD1)/GIGAS CELL (GIG) exit meiosis after the first division (Cromer et al. 2012; d’Erfurth et al. 2009). Studies of vegetative cells revealed that OSD1/GIG represses the action of CDC20 (Iwata et al. 2011). The analysis of CDC20 function is complicated by the presence of five presumably redundantly acting CDC20 genes in Arabidopsis. The simultaneous silencing of CDC20.1 and CDC20.2 via RNAi resulted in plants that produced very little pollen (Kevei et al. 2011). However, a detailed description of the function of CDC20 in the meiotic program is still pending.

Putative substrates of a meiotic APC/C^CDC20^ complex are TAM, the other being above-mentioned A-type cyclins as well as CYCB3;1. The two cyclins that are present throughout meiosis, CYCA3;3 and SDS, (Bulankova et al. 2013), lack or only have a degenerated destruction box, which serves as a recognition sequence for CDC20 (Glotzer et al. 1991). Interestingly, adding a destruction box prevents accumulation of SDS beyond pachytene similar to CYCA2;2, CYCA3;2 and CYCA3;4, which suggests that the APC/C becomes already active in mid prophase. In animals, cyclin A is also degraded before cyclin B in a yet not fully understood mechanism (Ramachandran et al. 2007; Fung et al. 2005). How a sequential turn-over of A- and B-type cyclins in plant meiosis is accomplished needs to be determined, but defined degradation steps could possibly contribute to the orchestration of the many different events necessary in meiosis.

## Meiotic checkpoints

Progression through the mitotic cycle is controlled at several transition points, also called checkpoints (Fig. 1). A first prominent checkpoint guards the entry into S phase (G1-S transition point) and requires that the activity of S-phase-specific CDK-cyclin complexes exceed a threshold level. A second checkpoint controls the entry into mitosis (G2-M transition point) and depends on M-phase-specific CDK-cyclin activity. Finally, a spindle checkpoint controls the activity of the APC/C and guards the metaphase–anaphase transition by assuring that all chromosomes are aligned on the equatorial plate and are attached to the mitotic spindle. In yeast and animals, several meiotic checkpoints have been identified that roughly correspond to these mitotic checkpoints. In contrast, meiotic checkpoints appear to be very differently setup in plants.

In yeast, the first meiotic checkpoint is the entry control into meiotic S phase (Fig. 1), for which the metabolic state of a cell appears to be of key importance as starvation induces meiosis (Egel 1971). While reports on meiotic arrests in plants are mounting, these seem of a different nature and not necessarily represent a checkpoint (see previous section on meiotic entry). In any case, entry into a meiotic program in plants does not appear to involve nutrient availability.

In animals and yeast, a meiosis-specific checkpoint, designated *meiotic recombination checkpoint*, is present at the end of pachytene stage permitting entry into diplotene stage only if the recombination process has been successfully completed (Roeder and Bailis 2000). By keeping the homologous chromosomes connected, properly processed crossovers are thought to allow the meiotic spindle to build up tension and this serves as a sign that the chromosomes can then be equally distributed to opposite poles of the cell (see also section on the APC/C) (Cooper and Strich 2011). The absence of this tension, as is presumably the case in mutants impaired in meiotic recombination such as mutants in the recombinase Dmc1 and homologous RecA family genes, triggers this pachytene checkpoint resulting in arrest of the meiotic program until spindle tension is established (Rockmill et al. 1995; Ghabrial and Schüpbach 1999; Takanami et al. 1998; Gartner et al. 2000; Odorisio et al. 1998). In mammals, a prolonged meiotic arrest can even lead to programmed cell death (Pittman et al. 1998; Yoshida et al. 1998). Without such a checkpoint, any absence of crossovers or failures in attaching the meiotic spindle to kinetochores would bear the risk of chromosome missegregation and subsequent aneuploidy resulting in severe developmental anomalies. Strikingly, a pachytene checkpoint appears to be not present or at least only in a much relaxed form in Arabidopsis since for example mutants in *DMC1* can complete meiosis (Couteau et al. 1999; De Muyt et al. 2009).

The pachytene checkpoint has been found to rely on many of the components of the mitotic DNA damage checkpoint (Wohlbold and Fisher 2009), and meiotic arrest is alleviated if mitotic DNA damage checkpoint components are inactivated (Lydall et al. 1996). In particular, the pachytene checkpoint has been found to depend on Wee1-type kinases, which catalyze phosphorylation of highly conserved Thr and/or Tyr residues in the P-loop of Cdk1-type kinases and by that block their activity (Berry and Gould 1996; Leu and Roeder 1999) (Fig. 2). Consistently, mutations in Cdc28, a Cdk1 homologue and the major CDK in budding yeast, cause arrest in pachytene (Shuster and Byers 1989). However, even in mutants that fail repairing meiotic DSBs in plants and hence suffer from massive chromosome fragmentation, the developmental program leading to the formation of gametes is typically completed. These mutants include *completion of meiotic recombination 1*/*sporulation in the absence of spo eleven 2* (*com1*/*sae2*)*, meiotic recombination 11* (*mre11*), *rad50,* and *rad51* (Uanschou et al. 2007; Gallego et al. 2001; Doutriaux et al. 1998; Li et al. 2004; Hartung and Puchta 1999; Bundock and Hooykaas 2002; Puizina et al. 2004).

The relaxed nature of the pachytene checkpoint in plants could at least be partially due to different mechanisms of how plants arrest the cell cycle after DNA damage. Although WEE1 homologues exist in plants and have for instance been isolated from maize, tomato, and Arabidopsis (Sun et al. 1999; Sorrell et al. 2002; Gonzalez et al. 2004), WEE1 function appears to have undergone functional diversification since at least in Arabidopsis, *wee1* mutants neither have mitotic problems nor are impaired to arrest the cell cycle after DNA double-strand breaks (De Schutter et al. 2007; Cools et al. 2011). Also, dephospho-mutants in CDKA;1 that cannot be phosphorylated by WEE1 are viable and not hypersensitive to DNA-damaging drugs (Dissmeyer et al. 2009, 2010). Consistently, recent observations suggested that instead of controlling cell cycle progression via CDKA;1, Arabidopsis WEE1 prevents premature cell differentiation after DNA damage in S phase in a yet unknown mechanism (Cools et al. 2011).

The last two major checkpoints in meiosis are the transition points from metaphase I to anaphase I and from metaphase II to anaphase II. In yeast and animals, these checkpoints resemble the mitotic spindle checkpoint that detects unattached chromosomes in order to prevent aneuploidy. However, at least the metaphase I to anaphase I checkpoint also does not appear to be very potent in plants since many mutants, i.e., in the above-mentioned *SPO11* or *DMC1* genes required for the induction, respectively processing of meiotic double-strand breaks, do not arrest anaphase I, but progress through meiosis and often lead to aneuploidy (Couteau et al. 1999; Hartung et al. 2007).

The less stringent meiotic checkpoints also appear to be relevant during wild-type plant development reflected by the relatively high number of spontaneous diploid pollen produced (De Storme and Geelen 2013; Brownfield and Kohler 2011). This has also evolutionary consequences as the formation of unreduced gametes is a major driving force in polyploidization and subsequently in speciation (Comai 2005; Kohler et al. 2010; Otto 2007). It is an interesting hypothesis that the occurrence of relaxed checkpoints is not only a byproduct of some not yet understood molecular mechanisms but may happen in a deliberate and controlled manner contributing to evolutionary plasticity of plants.

Remarkably, the metaphase I to anaphase I checkpoint appears to be differentially active in mammals since males but not, or least not very efficiently, females arrest meiosis after chromosomal misalignments, leading to chromosome aberrations such as trisomy 21 in humans (LeMaire-Adkins et al. 1997; Woods et al. 1999). There is also a not very well understood time component involved since the occurrence of chromosome aberrations increases with age of the females.

While proof for the existence of the meiotic checkpoints is missing in plants, we also still know only little about the kinetics of meiosis in the above-described mutants and there may be substantial delays in cell cycle progression that would implicate some checkpoint mechanisms in place. Furthermore, there may exist different control points not known in yeast or animals. For example, mutation of *am1* and *mel2* that disrupt a cells’ commitment to meiosis can cause early meiotic arrest in plants and lead the authors to suggest the presence of a meiotic leptotene/zygotene checkpoint (Che et al. 2011; Pawlowski et al. 2009) (see section above). Its nature might fundamentally differ from the DNA-damage-induced pachytene checkpoint as it is likely a consequence of pre-meiotic events (Nonomura et al. 2011). DUET/MALE MEIOCYTE DEATH1 (MMD1), a PHD finger protein, causes male meiocytes to arrest and undergo apoptosis at the end of meiotic prophase diakinesis/metaphase I (Reddy et al. 2003; Yang et al. 2003). It shows homology to MALE STERILITY1 (MS1), a transcriptional regulator of male gametogenesis the mutation of which causes arrest of microspore development (Ito et al. 2007; Wilson et al. 2001). Mutants of BLAP75 and Topoisomerase 3α (TOP 3α) that act together in the dissolution of homologous recombination intermediates cause arrest after chromosomes fragment at anaphase/telophase I (Chelysheva et al. 2008; Hartung et al. 2008). Fragmentation *per se* does not induce meiotic arrest, since *rad51* mutants, which also show fragmentation at meiosis I, progress through meiosis and can even rescue the TOP 3α telophase I arrest. Lastly, mutants in the presumptive-phosphoserine-binding protein SMG7 become arrested at the anaphase II to telophase II transition and are characterized by a failure to decondense chromosomes and reorganize the meiosis II spindle (Riehs et al. 2008). However, which of these various arrest points are genuine checkpoints or rather reflect the lack of an essential component necessary for the next step in the meiotic program needs to be determined in future.

## Exploiting the special features of the meiotic program in plants

The absence/low stringency of meiotic cell cycle checkpoints in plants offers an unprecedented possibility in breeding. Two novel breeding methods were recently shown to be feasible in plants (Arabidopsis) that make use of specific meiotic mutant situations to engineer new inheritance patterns. Notably, these mutant situations (a triple mutant of *spo11,* *rec8* and *osd1* or the RNAi-induced knock down of *DMC1*) would all have caused checkpoint-induced meiotic arrest in mouse (Romanienko and Camerini-Otero 2000; Bannister et al. 2004; Yoshida et al. 1998).

**Fig. 4:**
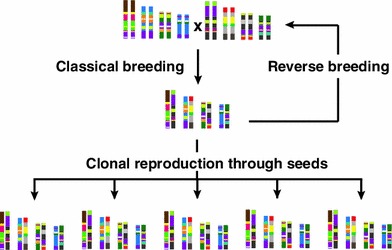
Relaxed meiotic checkpoints allow the development of new breeding approaches. Classical breeding refers to the classical method of constructing a hybrid by crossing two homozygous lines. Reverse breeding allows homozygous breeding lines to be constructed directly from a heterozygous parent essentially reversing classical breeding. Clonal reproduction through seeds allows the propagation of hybrids without homozygous intermediates. Please note that the given breeding schemes are simplified representations of these techniques. For further information please see Marimuthu et al. (2011), Wijnker et al. (2012) and Dirks et al. (2009)

Marimuthu et al. (2011) described the construction of a *spo11* *rec8 osd11* triple mutant in Arabidopsis in the F1 of a cross between two natural accessions (i.e., a plant homozygous for the mutations, but heterozygous for all other alleles present between the two accessions). In this triple mutant, no recombination occurs, sister chromatids segregate at meiosis I and the second meiotic division is omitted. Consequently, these plants execute a mitosis-like meiotic cell division that produces viable diploid spores with a genotype identical to the parent. Since in Arabidopsis, haploid or diploid gametes can directly be grown into seeds and subsequently into plants (Ravi and Chan 2010; Marimuthu et al. 2011), it was possible to grow offspring from this F1, which were identical to the mother plant, thereby effectively cloning the F1 through seeds (Marimuthu et al. 2011) (Fig. 4). Since contemporary breeding relies heavily on heterozygous varieties that are preferred because of their higher yield (Chen 2010), this modulation of meiosis may show a way how to propagate heterozygote crops as clonal lines rather than creating them anew each year by crossing homozygous parental lines.

A second proposed breeding method, reverse breeding, aimed at developing a technique to dramatically reduce the complexity of meiotic recombination, in which not alleles, but only non-recombinant parental chromosomes segregate (Wijnker et al. 2012). The authors showed the possibility to “deconstruct” a heterozygous genome into homozygous breeding lines carrying exactly half of the parental chromosomes. So when a potentially interesting, high producing heterozygous plant is encountered in an outcrossing population, it could be directly converted into homozygous (new parental) breeding lines (Fig. 4). In this case, a dominant RNAi-mediated knock down of the essential meiotic recombinase DMC1 abolished crossover formation in a heterozygous F1 and as a consequence resulted in missegregation of chromosomes leading to semi-sterility because of aneuploid gametes. However, the non-arrested progression of the meiotic program nevertheless ensures that cells in which—by chance—balanced chromosome segregation takes place, still produce viable gametes. These gametes containing non-recombinant chromosomes can be grown into haploid offspring, as mentioned above, and can subsequently be converted into homozygous diploid plants (so called doubled haploids). From among these doubled haploids, parental lines can be selected to reproduce the initially isolated hybrid. Reverse breeding is a potential versatile breeding tool that, apart from generating breeding lines for heterozygotes, could be used to produce chromosome substitution lines (Dirks et al. 2009).

## Conclusions

A detailed understanding of meiotic progression in plants is not only important for basic insights into one of the largest classes of living organisms and crucial components in most ecosystems on earth but also for emerging questions in speciation and genomic dynamics for which plants provide powerful model systems. The viability of many plant cell cycle and meiotic mutants furthermore allows the analysis of double mutants and the untangling of epistatic interactions, as was nicely illustrated through the experiments on the interactions between *smg7*, *tam* and *tdm*. The list of meiotic cell cycle regulators in plants is rapidly growing with major accomplishments just over the last few years. A main challenge in the future will be to untangle the specific from the redundant functions of the different CDK-cyclin complexes apparently at work in meiosis. Of key importance is the identification of the targets of these complexes and how their differential phospho-status will then promote the coordinated progression of the complex meiotic events. An immediate application of a deeper understanding of plant meiosis is the development of new plant-breeding strategies that may allow the propagation of hybrids and show the possibility to reconstruct complex allelic combinations. Thus, further insights into the regulation of the meiotic division program hold the promise for yet new possibilities in breeding to meet the challenges of our agriculture in the twenty-first century.
